# The Effectiveness, Facilitators, and Barriers of Digital Mental Health Services for First Nations People in Australia: Systematic Scoping Review

**DOI:** 10.2196/80386

**Published:** 2026-01-27

**Authors:** Siyu Zhai, Andrew Goodman, Anthony C Smith, Sandra Diminic, Xiaoyun Zhou

**Affiliations:** 1 School of Public Health, The University of Queensland, 266 Herston Road, Herston, Queensland, 4006, Australia, 61 452377835; 2 Australian eHealth Research Centre (AEHRC), Commonwealth Scientific and Industrial Research Organisation (CSIRO), Herston, Australia; 3 Centre for Online Health, The University of Queensland, Brisbane, Australia; 4 Hans Christian Andersen Children’s Hospital, Odense University Hospital, Odense, Denmark; 5 Centre for Innovative Medical Technology, University of Southern Denmark, Odense, Denmark; 6 Centre for Health Services Research, The University of Queensland, Herston, Queensland, Australia; 7 Queensland Centre for Mental Health Research, Brisbane, Australia

**Keywords:** digital mental health, Aboriginal and Torres Strait Islander people, eHealth, mHealth, mobile health, telehealth, mental health services

## Abstract

**Background:**

First Nations people in Australia experience inequitable mental health outcomes and service access. Digital mental health (DMH) services, which refer to offering mental health services through digital platforms, are considered potential solutions to address such mental health service inequities and improve the mental health outcomes of First Nations Australians. However, evidence on the effectiveness of DMH services for First Nations people in Australia is yet to be synthesized.

**Objective:**

This systematic scoping review aims to fill this gap and to identify the facilitators and barriers that influence the implementation of DMH services among First Nations people in Australia.

**Methods:**

A systematic search was conducted across 6 academic databases to search for studies related to DMH services for First Nations people in Australia. Search terms relating to First Nations people, geographic terminologies of Australia, mental health, and DMH services were used. Studies were included if they assessed the effectiveness of DMH services or the determinants of the facilitators and barriers of implementing DMH interventions among First Nations people in Australia. Data were extracted based on study design, targeted services, and research findings, and were then synthesized using a thematic analysis framework.

**Results:**

In total, 22 studies met the inclusion criteria. DMH services were used to support and treat First Nations Australians and conduct psychological assessments in these individuals. Evidence of effectiveness was stronger for nonsevere mental health conditions. The determinants of the facilitators and barriers of the implementation of DMH services included the following: (1) organizational and administrative factors; (2) cultural appropriateness; (3) accessibility; (4) integration of DMH services in the existing health system; (5) engagement between clients and service providers; (6) coverage of different conditions and clients; (7) acceptability to DMH services; (8) digital literacy; and (9) efficiency.

**Conclusions:**

Evidence on the use of DMH services for First Nations Australians remains heterogeneous in terms of study design and outcome measurement. DMH services appear to be most effective for managing nonsevere mental health conditions. Successful implementation requires multilevel structural support, including policy and organizational commitment, enhanced digital infrastructure, workforce training and engagement, and the design of culturally responsive DMH models to improve uptake and equitable access to mental health care among First Nations Australians.

## Introduction

Aboriginal and Torres Strait Islander people in Australia (“First Nations” people used hereafter) view mental health as a part of a holistic view of health across all life stages [1,2]. Maintaining a personal connection to traditional culture, health views, and community serves as an important protective factor for the mental health of First Nations people [1,3-6]. Therefore, reinforcing cultural strength and resilience would foster personal resilience and self-esteem, thereby protecting their individual mental health [7] and reducing the likelihood of developing mental illness [5,8,9]. Moreover, mental health services that acknowledge the right of self-determination and the need for cultural understanding of First Nations people [10], provide culturally appropriate care [11], or employ First Nations health workers [12] are also more likely to reinforce protective factors and achieve better treatment outcomes.

However, First Nations people are facing more complex mental health challenges than the general population in Australia due to external historical and social determinants [13]. Historically, colonization led to intergenerational trauma that worsened their mental health [14]. Currently, First Nations people continue to face daily discrimination [15], along with social inequity in areas such as education and employment [9]. These structural inequities increase the likelihood of mental health issues within these communities [16] and create mental health outcome gaps between First Nations people and the general population. For instance, although the national health survey showed that the prevalence of any mental illness among First Nations people dropped from 29.3% in 2014‐2015 [8] to 24% in 2018‐2019, it was still considered high [17]. Additionally, a survey conducted in regional Australia that applied structured clinical interviews in 2014‐2016 showed that the prevalence of any mental illness was 42.2% (4.2-fold higher than the prevalence in the general population) [5]. The prevalence of anxiety and mood disorders among First Nations people was estimated to be 1.6‐3.3 times the national prevalence in Australia [18]. Furthermore, 16% of deaths of First Nations people were related to suicide in 2001‐2005, which is 10% higher than the proportion in the general population [4].

Despite significant mental health needs and gaps, First Nations people experience barriers to accessing mental health services. First, structural racism, stigma toward mental health conditions, low health literacy, and less comfort in seeking help from health professionals prevent First Nations people from using more mental health services [11,12,19]. Second, a lack of local services and service providers creates barriers to travel for First Nations people [11,20], especially in areas with higher geographical remoteness that have a higher proportion of First Nations populations [21] but lower service access [22]. Moreover, community-controlled, culturally safe services may be unavailable for some First Nations people [23], and non-First Nations health workers who do not receive appropriate cultural safety training also contribute to service access barriers [20,24-26]. In 2018‐2019, only 31% of First Nations adults with high or very high levels of psychological distress received professional mental health services [27]. Moreover, mental health service gaps were mostly reported by First Nations primary health organizations (134 out of 198 organizations) [28]. Furthermore, among suicide cases, First Nations populations were approximately 2 times less likely than non-First Nations populations to have received mental health support (23.8% vs 43.3%) [29]. In the Northern Territory, mental health service utilization of First Nations youth is nearly half that of non-First Nations youth (1.9% vs 4.1%) [30].

Digital mental health (DMH) services refer to providing mental health services through digital technologies [31-33]. DMH services have been implemented for the prevention, screening, intervention, and rehabilitation of mental health conditions [34] with or without mental health professionals’ guidance or support [35], and they demonstrate promising results for managing various mental health conditions, including anxiety disorders [36], depressive disorders [37], posttraumatic stress disorder [38], and alcohol and other drug-related mental disorders [39]. Moreover, DMH services have been evaluated among different populations, including children and young people [40], adults [41], and older populations [42].

DMH services have been evaluated among First Nations people worldwide [43-46] and have shown the potential to address the current health outcome gaps faced by them. Evidence from Canada, New Zealand, and the United States indicates that DMH services support improvements in psychological assessment [47-50] comparable to existing face-to-face services. DMH services were also found to improve clinical outcomes [49,51] and continuity in mental health care [52], both as stand-alone and blended care. International evidence also suggests that DMH services can reduce mental health gaps by improving the accessibility and availability of mental health services among First Nations people [23,34-36].

In Australia, evidence on DMH services among First Nations people is mixed. Although digital gaps remain a barrier to DMH implementation [53], government funding has enabled the provision of digital infrastructure in rural and remote First Nations communities [54]. Another service gap is the need for culturally appropriate services [23]. Evidence suggests that DMH services may fail to deliver culturally safe services [55,56] due to the preference of First Nations people for the familiarity and safety of face-to-face services [50]. However, some studies have indicated that digital health may support cultural safety by increasing the involvement of First Nations health workers [43,57] and community-led design services [58], and by allowing patients to see familiar faces via video conferencing [54]. DMH services also enable First Nations people with mental health needs to include family and community members in decision-making, without the need for travel [54].

Currently, most systematic reviews have focused on international evidence regarding the application of DMH services to First Nations people [43-46]. Existing Australian evidence is more likely to review broad and general health conditions rather than focus on mental health [47,54,57,59] or to solely focus on the design and assessment of DMH services for First Nations people rather than the implementation [58]. There is a paucity of evidence on the effectiveness of DMH services and the facilitators and barriers of implementing these services among First Nations people in Australia. Our study aims to address this research gap by exploring the following research questions: (1) What is the effectiveness of DMH services for First Nations people in Australia? (2) What are the determinants of the facilitators and barriers of implementing DMH services among First Nations people in Australia? Such evidence is critical for policymakers, health providers, and First Nations users to make evidence-informed decisions while funding, implementing, prescribing, and choosing DMH services.

## Methods

### Reporting and Registration

The reporting of this study followed the PRISMA (Preferred Reporting Items for Systematic Reviews and Meta-Analyses) 2020 guidelines (Checklist 1) and was registered through PROSPERO (CRD42024612517).

### Acknowledgment to Indigenous Data Sovereignty

This study follows the Maiam Nayri Wingara principles [60]. The authors acknowledge that First Nations people have control of the data ecosystem; all data are available to First Nations people; and this study pays respect to First Nations communities, will be accountable to these communities, and empowers the sustainable self-determination of these communities.

### Outcome Definition

Our study outcomes include the effectiveness of DMH services and the determinants of facilitators and barriers. The effectiveness of DMH services is defined as the extent to which DMH services obtain the desired mental health outcomes. For example, for DMH services that are related to psychological diagnosis and assessment [49], the accuracy of diagnosis is considered as effectiveness. For treatments provided through DMH services [51,52,61], effectiveness is defined as the degree to which a treatment service improves mental health symptoms. The determinants of facilitators and barriers are defined as the determinants that can promote and impede, respectively, the implementation of DMH services for First Nations people in Australia.

### Eligibility Criteria

Based on the PICO (population, intervention, comparison, outcome) protocol, this review considered studies that (1) included participants who were First Nations Australians; (2) included interventions that were DMH services with various functions; (3) included comparisons that were typical mental health services or did not include comparisons; and (4) targeted outcomes that involved changes in mental health conditions. In addition, studies written in English were included. Studies that were irrelevant to our PICO criteria and those that did not have full text available were excluded. Moreover, there was no eligibility criterion relating to First Nations groups, date of publication, or age of participants. See Multimedia Appendix 1 for detailed inclusion and exclusion criteria and the PICO protocol.

### Search Strategy

A systematic search was conducted from September 9, 2024, to September 15, 2024, across 6 online databases: PsycINFO, PubMed, MEDLINE, Embase, Web of Science, and Google Scholar. Considering that more than 10,000 search results were obtained from Google Scholar, searching on this database was limited to the first 30 pages.

The search strategy was developed after consultation among 3 authors (SZ, XZ, and SD). Boolean operators, truncations, and search terms were tailored to each database. The search terms were organized around the following themes: First Nations people, geographic terminologies of Australia, mental health (including specific mental health conditions), and DMH services. Considering that DMH is a relatively new research area, there was no limitation on the publication date of the searched studies. See Multimedia Appendix 2 for details of the search strategy.

### Study Selection

Selected studies were imported into EndNote 20 and Covidence, and duplicates were removed at this stage. Afterwards, the titles and abstracts were screened. Studies found to be irrelevant to the review topic based on an assessment of the title and/or abstract were excluded. Studies that had insufficient information in the title and abstract were moved to full-text screening. During full-text screening, studies that met the exclusion criteria were excluded.

One reviewer (SZ) conducted the title and abstract screening. Uncertainty in this process was solved through discussion with another reviewer (XZ), and any disagreements were resolved by involving the third reviewer (SD). Two reviewers completed the full-text screening, where one reviewer (SZ) made initial decisions and the other reviewer (XZ) checked the findings. Therefore, the selection process was not blinded. Disagreements were resolved through discussion or by involving the third reviewer (SD).

### Data Extraction

Extracted data were tabulated based on author, year of publication, study design (qualitative, quantitative, or mixed methods), study aim, study population, research setting, type of DMH intervention, outcome (effectiveness, facilitators, and barriers), and key research findings. See Multimedia Appendix 3 for details of the extracted data.

For missing data, if the authors did not specify the reason, the reviewers tried to contact the authors to obtain relevant information. If such information was unavailable or no response was received, the missing data were excluded from the analysis process.

### Strategy of Data Synthesis

The synthesis of data followed the thematic analysis method introduced by Thomas and Harden [62] and was guided by the SWiM (Synthesis Without Meta-Analysis) guideline [63].

Reviewer SZ read selected studies completely and focused on the results section of those studies. Results relevant to the targeted outcomes of this review, such as the effect size of interventions or reported user experiences related to these determinants, were summarized and coded into descriptive themes. Descriptive themes were categorized into subgroups and synthesized based on the type of data (qualitative or quantitative), research design (with or without a control group), and relevant determinants (facilitators, barriers, or effectiveness). Afterwards, reviewers standardized themes using the direction of the effect. Vote counting was conducted based on the direction of the effect. Then, the commonality among descriptive themes was found based on the direction of the effect or the different determinants they related to, which were used to formulate the analytical themes that summarized multiple similar descriptive themes. Analytical themes interpreted the primary results to fit the objectives of this review. To ensure the trustworthiness and validity of thematic analysis, during the generation of themes, reviewer SZ consulted reviewers XZ and SD to resolve any uncertainty. Once reviewer SZ completed generating themes, reviewer XZ checked all themes. Any disagreements were resolved by consulting the third analyst, SD.

### Quality Assessment

This study applied the Aboriginal and Torres Strait Islander Quality Appraisal Tool [64] to assess the quality of health research from a cultural sensitivity perspective. Reviewer SZ carefully reviewed the included studies and answered the 14 questions in the Aboriginal and Torres Strait Islander Quality Appraisal Tool based on the content of all selected studies. Moreover, as the instructions for this quality assessment tool suggested, the numbers of studies assessed as “yes,” “partially,” “no,” and “unclear” have been reported. Reviewer SZ applied the Mixed Methods Appraisal Tool (MMAT) [65] for assessing the methodological quality of the included studies. Only the relevant part of the included study was assessed. For instance, if the qualitative part of a mixed-methods study was the only part relevant to this review, the study was appraised as a qualitative study. The MMAT does not recommend scoring each study; hence, this review only reports the performance of the included studies on each criterion and the overall trend of the design of the included studies. All quality assessment results were checked by reviewers XZ and SD. See MultimediaAppendices 4  5 and the Results section for the quality assessment results.

In this systematic scoping review, all studies were included regardless of quality rating. However, the quality appraisal results informed the interpretation of the searched studies. Considering this study was a narrative synthesis and did not include a meta-analysis, no statistical weighting or sensitivity analysis was performed.

## Results

### General Characteristics of the Studies

**Figure 1.**
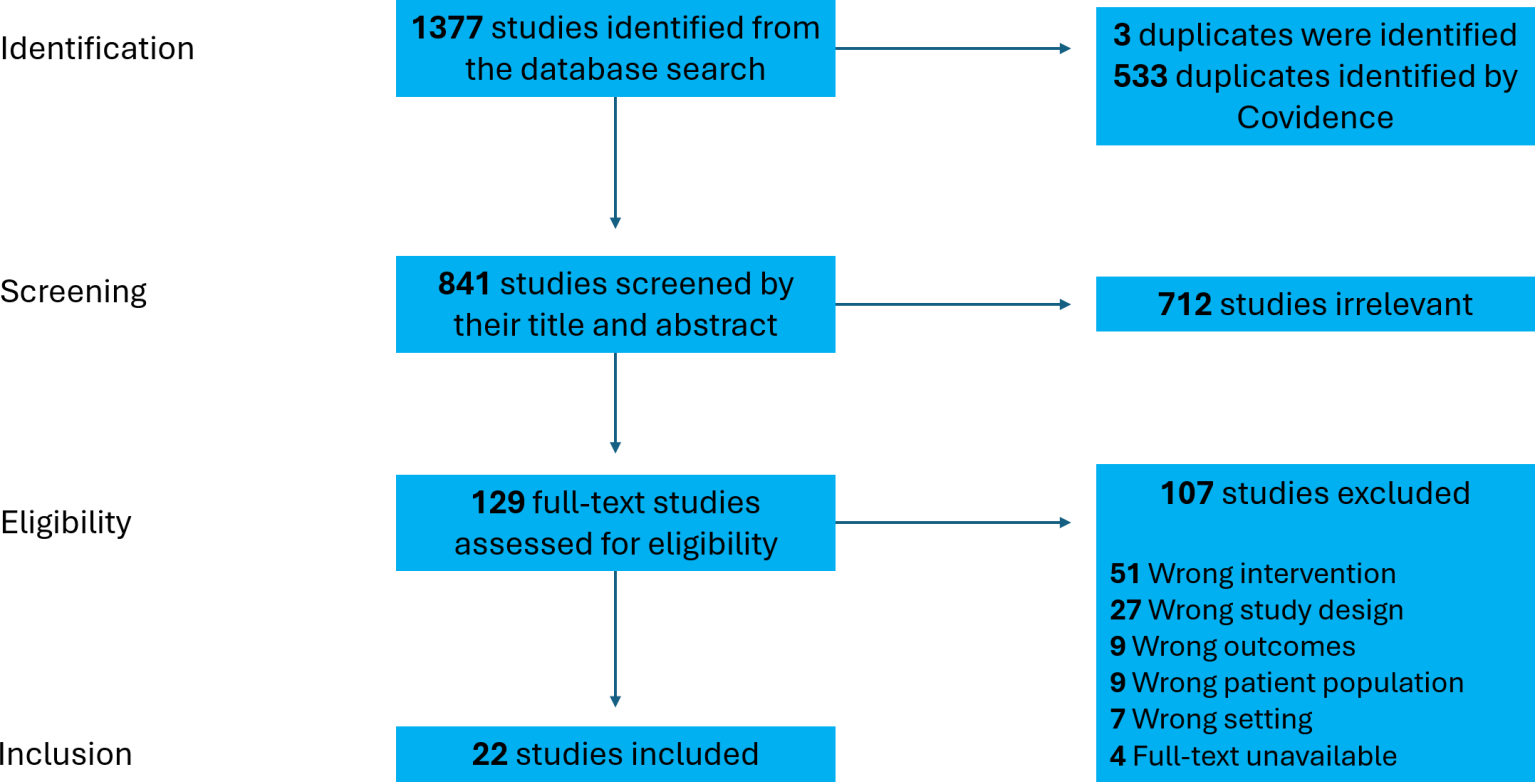
PRISMA (Preferred Reporting Items for Systematic Reviews and Meta-Analyses) flowchart of article screening and inclusion.

A total of 22 articles were included in this review [66-87]. The PRISMA flowchart is presented in Figure 1. See Multimedia Appendix 3 for detailed characteristics. Except for 1 study that solely reported barriers [73] and 4 studies that solely reported effectiveness [72,82,85,87], the rest of the studies reported more than one targeted outcome (ie, facilitators, barriers, and effectiveness). Moreover, 16 studies [67,68,71-74,76,77,79-84,86,87] were published within 5 years, and all included studies were published between 2015 and 2023.

Among the included studies, there were 8 qualitative studies [66,67,69,74,75,78,79,83], 8 mixed-method studies [68,70,71,76,77,80,81,84], and 6 quantitative studies [72,73,82,85-87]. Among the quantitative studies, 3 adopted a cross-sectional research design [72,73,87], 1 was a crossover study [82], 1 was a randomized controlled trial [85], and 1 was a prospective cohort study [86].

All studies involved First Nations people as the clients of DMH services. There were 5 studies involving health care providers who provided DMH services to First Nations people [66,69,78-80], 2 studies involving both clients and service providers [67,74], 12 studies involving adult clients or clients whose ages were unspecified [68,70-73,75,81,82,84-87], and 3 studies involving youth or student clients [76,77,83].

Regarding the types of DMH services, 5 studies were related to general perspectives on DMH services [66,77-80]. Among the studies, 2 involved DMH services delivered through web pages [70,86], 12 involved DMH services delivered through mobile apps [67-69,71,72,74-76,79,80,84,85], 5 involved DMH services delivered through telehealth methods (text or voice-only phone call) [70,73,86,87], 1 involved DMH services delivered through video conferencing [82], and 2 involved DMH services containing both online and offline activities [81,83]. See Table 1 and Multimedia Appendix 3 for the details of DMH service types.

**Table 1.** Overview of the findings in relation to the effectiveness of digital mental health services.

Evidence type and study (author, year)	Data collection method	Data analysis method	Digital mental health service type	Major findings
Qualitative evidence				
Dingwall et al [68], 2023	Qualitative interviews at the end of the 4-week intervention	Deductive approach for qualitative analysis	Aboriginal and Islander Mental Health Initiative for Youth (AIMhi-Y) app	Participants had mental health improvements or gained help during tough times by using this app, which suggested its effectiveness.
Perdacher et al [74], 2022	Semistructured interviews	Thematic analysis under the framework provided by the constant comparison method	Stay Strong app (custodial version)	Both clients and practitioners reported that the app improved the abilities of self-insight and self-reflection of clients and guided the positive thinking of clients. Both clients and practitioners reported that the app could enhance clients’ confidence, view of self, and empowerment.
Povey et al [75], 2016	Three focus groups	Thematic analysis	AIMhi Stay Strong iPad app and the iBobbly suicide prevention app	Both apps have the potential to help people with minor mental health conditions overcome difficulties. For the suicide prevention function, participants thought the app is useful for nonsevere conditions but may not be helpful in preventing suicide due to it being a severe condition.
Raphiphatthana et al [80], 2020	Semistructured interviews	Thematic analysis that oscillated between deductive and inductive approaches	General digital mental health services (but many of the responses were related to the use of the Stay Strong app)	Electronic mental health was perceived as potentially beneficial and useful for First Nations communities.
Routledge et al [81], 2022	Paper-and-pen test in classes or an online questionnaire	Thematic analysis	Web-based lesson package, Strong & Deadly Futures	Some facilitators reported that students learned the knowledge that the lesson planned to deliver, while students reported that the substance-related knowledge had been learned and understood.
Tighe et al [84], 2020	Semistructured interview	Thematic analysis	iBobbly program	Participants indicated that an app may not be able to provide enough support for vulnerable people who have severe mental health conditions. The improved mental health literacy helped participants feel distracted from their thoughts and reduced their distress. Some participants reported that the program has the effect of suicide prevention.
Quantitative evidence with comparison to typical mental health services or no intervention groups				
Lee et al [72], 2019	Intervention group: data from the Grog Survey app; Control group: clinical interview	Triangulating the Finnish method to compare drinking status and average consumption between 2 groups using descriptive statistics. Agreement between consumption data/daily alcohol consumption and withdrawal tremors was assessed through the Spearman rank correlation coefficient. Sensitivity and specificity analyses (unspecified) were used to compare how well the app and clinical interview agreed when classifying drinkers’ risk.	Grog Survey app	The app can accurately test participants’ drinking status (n=157), with 94.7% agreement with the clinical interview results. The alcohol consumption level recorded by the app was usually higher than clinical interviews (drinks per drinking occasion: median 17.0, IQR [10.5, 27.9] vs median 15.4, IQR [9.6, 23.2]). The app tended to categorize more participants at risk. The app identified 76.7% of drinking males and 69.7% of females as being at risk, while the interview identified 74.4% of males and 65.2% of females as being at risk. The app appeared to be highly sensitive (92.7%‐97.5% sensitivity) in detecting at-risk drinkers, and when using matched and unmatched reference periods, the app could detect 10 and 37 more risky drinkers, respectively, than clinical interviews. The average daily consumption recorded by the app and by the interview was moderately correlated (*r*=.52). Correlations were greater when only data from matched time periods were used (*r*=.62). Correlations were all statistically significant. Participants were more likely to report the presence of tremors when using the app than when responding to the interview (17.0% and 7.2%, respectively; *r*_s_=0.48). There was high test-retest reliability for the app (*r*_s_=0.81 for both participants and drinkers).
Veinovic et al [87], 2023	Test results of the face-to-face and telehealth versions of both the MMSE^a^ and the KICA-screen^b^ tests	The MMSE and KICA-screen test version total scores were compared using Spearman correlations. Agreement between test versions was examined using Bland-Altman analyses. Telephone test scores were prorated to equivalent in-person total scores to examine overall performance discrepancies directly.	Telehealth versions of the MMSE (TMMSE) and KICA-screen (TKICA-screen)	Despite a reduction in effectiveness, the accuracy of the TMMSE was generally acceptable. The median TMMSE results were slightly lower than the MMSE results (24 vs 28.5, IQR 2 for both). There was a moderate correlation and a reasonable agreement between MMSE versions (*r*_s_=0.33; *P*=.20), although the limits of agreement were unacceptably wide (−4.1 and 4.8 point differences). The TKICA-screen was significantly less effective than the KICA-screen. There was a significant mean difference of 2.17 (95% CI 1.39‐2.95), with wide limits of agreement (−1.09 and 5.43 point differences) and evidence of proportional bias (B=−1.03, SE=0.32; *P*=.005), with a tendency for poorer agreement at the lower end of the performance.
Tighe et al [85], 2017	Test results from the following psychological instruments: Depressive Symptom Index-Suicidality Subscale (DSI-SS), Patient Health Questionnaire-9 (PHQ-9), Kessler Psychological Distress Scale-10 (K-10), and Barratt Impulsivity Scale-11 (BIS-11)	Paired *t* test	iBobbly program	The effectiveness of the iBobbly app on suicide prevention was insignificant. The pre- and postintervention changes in the DSI-SS score were significant in the iBobbly arm (*t*=2.40, df=58.1; *P*=.02), but these differences were not significant compared with the waitlist arm (*t*=1.05, df=57.8; *P*=.30). The iBobbly app was effective in reducing depression. The iBobbly arm showed a statistically significant decrease in PHQ-9 scores compared with waitlist controls. The interaction of the iBobbly arm by time was significant (*t*=2.79, df=56.9; *P*=.007; Cohen *d*=0.71, 95% CI 0.17-1.23), reflecting a substantial effect. The iBobbly app was effective in reducing general psychological distress measured by the K-10. The interaction of the intervention arm by time was significant (*t*=2.44, df=57.5; *P*=.02; Cohen *d*=0.65, 95% CI 0.12-1.17), reflecting a substantial effect. However, the PHQ-9 and K-10 scores for the control group (comparing baseline and receiving the iBobbly intervention afterwards) were insignificant. The iBobbly app was not effective in reducing impulsivity, as measured by the BIS-11. The preintervention scores for the waitlist group were significantly lower than those for the iBobbly group (*t*=2.05, df=59.2; *P*=.045). Postintervention means were identical. However, after applying the iBobbly app to the waitlist group, the score reduction was insignificant (*t*=−1.82, df=29.1; *P*=.08).
Russell et al [82], 2021	KICA-screen results obtained from both face-to-face and online tests	Liner correlation	KICA-screen delivered via telehealth	The KICA-screen test delivered through digital platforms did not cause a decrease in effectiveness compared to the face-to-face version, owing to good correlation (Pearson *r*=0.851; *P*<.01) and good agreement (intraclass correlation coefficient=0.85; *P*<.01).
Titov et al [86], 2019	Health records measured by the K-10, PHQ-9, and Generalized Anxiety Disorder Scale-7 (GAD-7)	A generalized estimation equation (GEE) modeling technique was used to examine the significance of changes in symptom measures over time. The main effects of course and Indigenous status were also examined.	MindSpot service	For First Nations patients, the MindSpot service was effective at causing a significant decrease in the K-10 score (*χ*^2^=5933.1; *P*<.001), PHQ-9 score (*χ*^2^=5938.8; *P*<.001), and GAD-7 score (*χ*^2^=5658.8; *P*<.001). There were no differences in treatment outcomes between Indigenous and non-Indigenous patients (K-10: *χ*^2^=0.1; *P*=.75; PHQ-9: *χ*^2^=0.5; *P*=.49; GAD-7: *χ*^2^=0.6; *P*=.44). There was no difference in the outcome between Indigenous patients who chose the general wellbeing course and those who chose the Indigenous wellbeing course (K-10: *χ*^2^=1.2; *P*=.27; PHQ-9: *χ*^2^=0.5; *P*=.48; GAD-7: *χ*^2^=2.9; *P*=.09).
Quantitative evidence with no comparison				
Dingwall et al [68], 2023	Comparing participants’ scores of psychosocial instruments at baseline and after the 4-week intervention: K-10, Patient Health Questionnaire-2 (PHQ-2), Generalized Anxiety Disorder-short form (GAD-2), Alcohol Use Disorders Identification Test (AUDIT-C), Drug Use Disorders Test (DUDIT), and Parent-rated Strengths and Difficulties Questionnaire (SDQ-parent rated)	Paired sample 2-tailed *t* tests for psychological test results with within-group effect sizes (Cohen *d*)	AIMhi-Y app	The app was significantly effective in reducing psychological distress (*t*_29_=4.63; *P*<.001; Cohen *d*=0.85, 95% CI 2.79-7.21) and depressive symptoms (*t*_29_=4.09; *P*<.001; Cohen *d*=0.75, 95% CI 0.42-1.25). Participants presented improvements in reducing anxiety symptoms (*t*_29_=1.58; *P*=.13; Cohen *d*=0.28, 95% CI −0.10 to 0.77). The app was ineffective in reducing alcohol use disorder symptoms (*t*_26_=1.55; *P*=.13; Cohen *d*=0.31, 95% CI −0.06 to 0.43), drug use disorder symptoms (*t*_26_=1.55; *P*=.13; Cohen *d*=0.31, 95% CI −0.06 to 0.43), and lead dependence (*t*_29_=0; *P*>.99; Cohen *d*=0, 95% CI −0.29 to 0.29). Participants’ parents reported that the app was ineffective in improving psychological adjustment (*t*_3_=2.15; *P*=.12; Cohen *d*=1.07, 95% CI −2.03 to 10.53).
Kennedy et al [71], 2021	The uMARS^c^ survey comprises 26 questions and 5-point Likert scales	Calculate descriptive statistics and mean and SD for continuous variables	Multibehavioral change app “MAMA-EMPOWER”	Participants (n=16) reported acceptable perceived impact (mean 3.55, SD 1.06).
Tighe et al [84], 2020	Record usage of the app and results from psychological instruments: DSI-SS, K-10, and PHQ-9	Regression analyses	iBobbly program	The iBobbly app was ineffective for the primary outcome, suicidal ideation, which was measured with the DSI-SS. For every minute spent on the app, DSI-SS scores reduced by 0.013 points (R^2^=0.29). The iBobbly app was ineffective in reducing psychological distress that was measured with the K-10. For every minute spent on the app, K-10 scores reduced by 0.007 points (R^2^=0.35). The iBobbly app was ineffective in reducing depression that was measured using the PHQ-9. For every minute spent on the app, PHQ-9 scores reduced by 0.001 points (R^2^=0.268). According to the authors, the low validity could be caused by the small sample size.
Routledge et al [81], 2022	Paper-and-pen test on classes or an online questionnaire	Analysis of descriptive data (method unspecified)	Web-based lesson package, Strong & Deadly Futures	Among First Nations students (n=15), more than half (53.3%) reported that they were likely to use the information and skills taught by the course in their own lives. Nearly half (46.7%) of First Nations students were unsure, and 0% of First Nations students believed they were unlikely to use the information and skills taught by the course. First Nations students generally thought this program was effective in handling problems related to substance use (73.3% of students supported this idea), stress (66.7%), and peer pressure (80%).

a MMSE: Mini-Mental State Examination.

b KICA-screen: Kimberly Indigenous Cognitive Examination-short form.

c uMARS: user version of the Mobile Application Rating Scale.

### Targeted Mental Health Conditions

Five studies did not specify the mental health conditions targeted by DMH services [66,67,70,77-80]. Three studies focused on psychological assessment and diagnosis [82,86,87], and 5 studies addressed the management of distress, depression, and anxiety disorders [71,75,84-86]. Additionally, 5 studies examined the management of substance use [71,72,75,76,83], and another 5 studies were related to the management of suicide or self-harm behaviors [67,73,75,84,85]. See Table 2 for detailed information about the settings of the included studies.

**Table 2.** Settings of the included studies.

Study (author, year)	Study aim	Participants	Research setting	Digital mental health (DMH) service type	Targeted mental health issue
Bennett-Levy et al [66], 2017	Report the barriers and enablers of e-mental health uptake among health providers who provide services to First Nations people	50 providers of different mental health services for First Nations people	Two locations (Lismore and Tweed Heads) in northern New South Wales	General e-mental health services (did not specify service type)	Unspecified
Brown et al [67], 2020	Understand how mobile apps support suicide prevention gatekeepers in First Nations communities	12 participants (Indigenous health workers or community members)	A regional city in South West Queensland called Toowoomba	Suicide prevention efforts supported by mobile apps	Suicide and self-harm behaviors
Dingwall et al [68], 2023	Assess the feasibility, acceptability, and use of the Aboriginal and Islander Mental Health Initiative for Youth (AIMhi-Y) app and determine the feasibility of study procedures for future assessments	30 First Nations young people aged 12‐25 years	Darwin, Northern Territory (NT)	AIMhi-Y app	Unspecified
Dingwall et al [69], 2015	Develop and determine the acceptability, feasibility, and appropriateness of the Australian Integrated Mental Health Initiative (AIMhi) Stay Strong App for service providers working with First Nations people	15 service providers, including health professionals, managers, program coordinators, and an Aboriginal elder	Rural and remote primary health care service settings in the NT	AIMhi Stay Strong app	General mental health area (unspecified)
Fletcher et al [70], 2017	Test the acceptability and feasibility of developing the “Stayin’ on Track” website, which offers tailored support and information to young Aboriginal fathers, and adapt and test the mobile phone–based text-messaging and mood-tracker program (SMS4dads program) that provides mental health support to young Aboriginal fathers	20 young Aboriginal fathers in the assessment of the “SMS4dads” smartphone program; 20 young Aboriginal fathers and an uncertain number of community members in the yarn-up session on the “Stayin’ on Track” website	One regional city and two rural towns in New South Wales	“SMS4dads” smartphone program and “Stayin’ on Track” website	General mental health area (unspecified)
Kennedy et al [71], 2021	Describe the development (stages 1 and 2) and pretest (stage 3) of a prototype multibehavioral change app (MAMA-EMPOWER)	Stage 1: 8 First Nations women; stage 2: 6 First Nations women; stage 3: 16 First Nations women	Newcastle and Tamworth, New South Wales	Multibehavioral change app “MAMA-EMPOWER”	Substance use and psychological distress
Lee et al [72], 2019	Validate a survey app (Grog Survey app) that explores the alcohol consumption status among First Nations people	20 nondrinkers, 40 nondependent drinkers, and 40 dependent drinkers who self-identified as First Nations people and were aged ≥16 years	An Indigenous primary health care service and surrounding community in urban Queensland, and a regional Aboriginal community–controlled health service and a remote Aboriginal community–controlled drug and alcohol day center (a drop-in service) in South Australia	Grog Survey app	Substance use (alcohol consumption)
Ma et al [73], 2022	Understand the expectations of different communities for telehealth crisis support services in Australia	1300 adults living in Australia, including 2.4% First Nations participants	National setting in Australia	Lifeline Australia	Suicide and self-harm behaviors
Perdacher et al [74], 2022	Determine the feasibility of the Stay Strong app as a digital well-being and mental health tool for use by First Nations people in prison	27 clients and 10 health practitioners	3 high-security prisons in Queensland, Australia	Stay Strong App (custodial version)	General mental health area (unspecified)
Povey et al [75], 2016	Explore First Nations community members’ experiences of using 2 e-mental health apps and identify the factors that influence acceptability	9 First Nations adults	Darwin, NT	AIMhi Stay Strong iPad app and the iBobbly suicide prevention app	AIMhi Stay Strong app: general mental health issues and substance use; iBobbly app: psychological distress, depressive symptoms, and suicide prevention
Povey et al [76], 2022	Present an in-depth account of the second phase of participatory design in the development of the AIMhi-Y app	75 First Nations youth	Across Australia	AIMhi-Y app (under design)	General mental health area (unspecified)
Povey et al [77], 2020	Report the result of the formative stage of the AIMhi-Y app development process that engaged First Nations youth in the co-design of the new culturally informed AIMhi-Y app	45 First Nations youth	NT, Australia	General e-mental health services (did not specify service type)	General mental health area (unspecified)
Puszka et al [78], 2016	Understand stakeholder perspectives on the requirements for implementing DMH services in regional and remote health services for First Nations people	32 stakeholders who provide mental health services for First Nations people	NT, Australia	General e-mental health services (did not specify service type)	General mental health area (unspecified)
Raphiphatthana et al [79], 2020	Evaluate the process and effectiveness of the e-mental health program	66 participants from First Nations primary care organizations	Remote NT communities, Darwin, Alice Springs, and Adelaide	Not specified, but most participants used the Stay Strong app	General mental health area (unspecified)
Raphiphatthana et al [80], 2020	Understand service providers’ perspectives and experiences of electronic mental health adoption	57 service providers working with Aboriginal and Torres Strait Islander people who had undergone electronic mental health training workshops	Darwin, Alice Springs, and remote NT communities	General DMH services (but many of the responses were related to the use of the Stay Strong app)	Unspecified
Routledge et al [81], 2022	Assess the acceptability and feasibility of Strong & Deadly Futures, a culturally inclusive alcohol and drug prevention program for First Nations secondary students	19 First Nations students out of 281 students	Two independent urban schools and two rural state schools	Strong & Deadly Futures, a 6-lesson, web-based package combining online illustrated storylines with interactive classroom activities	Substance use (both alcohol and tobacco)
Russell et al [82], 2021	Examine the utility of using a culturally appropriate dementia screening tool (KICA-screen^a^) in a telehealth setting	33 medically stable First Nations inpatients/outpatients	Two local rural health care settings	KICA-screen delivered via telehealth	Cognitive assessment
Snijder et al [83], 2021	Describe the development of Strong & Deadly Futures, a web-based substance use education class	41 First Nations students and 36 non-First Nations students	Four schools in New South Wales	Strong & Deadly Futures, a 6-lesson, web-based package combining online illustrated storylines with interactive classroom activities	Substance use (both alcohol and tobacco)
Tighe et al [85], 2017	Evaluate the effectiveness of a self-help mobile app (iBobbly) targeting suicidal ideation, depression, psychological distress, and impulsivity among Indigenous youth in remote Australia	61 First Nations Australians aged 18‐35 years	Remote and very remote communities in the Kimberley region of North Western Australia	iBobbly program	Suicidal ideation, depression, psychological distress, and impulsivity
Tighe et al [84], 2020	Discover pilot usage and acceptability data from the iBobbly suicide prevention app, an app distributed through a randomized controlled trial	13 First Nations Australians aged 18‐35 years	Remote and very remote communities in the Kimberley region of North Western Australia	iBobbly program	Suicidal ideation, depression, psychological distress, and impulsivity
Titov et al [86], 2019	Report on Aboriginal and Torres Strait Islander (Indigenous) users of MindSpot, a national service for the remote assessment and treatment of anxiety and depression	780 First Nations participants	National setting across Australia	MindSpot service, including diagnosis and treatment courses	General mental health area, but this study focused on psychological distress, depression, and anxiety
Veinovic et al [87], 2023	Evaluate mental state examinations delivered face-to-face (MMSE^b^ and KICA-screen) and as telehealth versions (TMMSE^c^ and TKICA-screen^d^) among older First Nations people	20 First Nations people aged 55‐69 years	Urban and regional New South Wales, Australia	TMMSE and TKICA-screen	Cognitive assessment

a KICA-screen: Kimberly Indigenous Cognitive Examination-short form.

b MMSE: Mini-Mental State Examination.

c TMMSE: telehealth version of the MMSE.

d TKICA-screen: telehealth version of the KICA-screen.

### Quality Assessment Results

According to the results from the Aboriginal and Torres Strait Islander Quality Appraisal Tool, the included studies presented an acceptable connection to First Nations values and principles, with at least 16 studies having “Yes” or “Partially” (provided sufficient information) answers to 11 out of 14 questions. However, most studies (n=14-18) did not properly negotiate a formal agreement with First Nations people to access and protect their knowledge. Moreover, some studies were deemed less culturally engaging for First Nations people, such as a study that researched DMH services designed for the general population [73], and some studies applied quantitative methods that resulted in lower participation in First Nations communities [82,86,87].

In terms of the results from the MMAT, all studies were considered to have high-quality research designs. For example, all qualitative studies, except for 1 study [71], completely matched all MMAT criteria. Moreover, all included studies had at least 82% of the criteria marked as “Yes.”

### Effectiveness

Twelve studies [68,71,72,74,75,80-82,84-87] reported the effectiveness of DMH services in managing mental health conditions among First Nations people in Australia (Table 1). Their results are summarized in the following paragraphs based on their study designs.

#### Qualitative Evidence

Six studies used a qualitative study design to explore the perceived effectiveness of DMH services in managing mental health conditions among First Nations people [68,74,75,80,81,85]. Three studies [68,74,75] assessed the youth version, custodial version, or standard version of the Aboriginal and Islander Mental Health Initiative (AIMhi) Stay Strong app for First Nations people. The AIMhi Stay Strong app helped participants achieve mental health improvements, guided positive thinking, and supported participants in overcoming difficulties [68,74,75]. Participants perceived the AIMhi Stay Strong app and general DMH services to be effective for improving their mental health outcomes [80]. Moreover, “Strong and Deadly Futures” online courses were examined among First Nations people to prevent alcohol and tobacco use and reduce stress and peer pressure [81]. Both clients and practitioners reported that the app enhanced clients’ self-insight, self-reflection, positive thinking, confidence, and overall self-perception. However, in the study by Povey et al [75], while both the AIMhi Stay Strong and iBobbly apps were viewed as helpful for managing minor mental health difficulties, participants considered them less suitable for addressing severe conditions or preventing suicide. Participants agreed that the efficacy of the iBobbly app was limited for “vulnerable people with strong emotions” [84].

#### Quantitative Evidence

Eight studies reported quantitatively measured effectiveness [68,71,72,82,84-87], with mental health outcomes measured through self-reported instruments and scales.

#### Quantitative Evidence Without Comparison

Four studies reported the effectiveness of DMH services in managing mental health conditions among First Nations people without comparing them to typical services or other control groups [68,71,81,84]. Kennedy et al [71] found that the multibehavioral change app “MAMA-EMPOWER” was effective in managing substance use and psychological distress and was acceptable (average score of 3.55 on a 5-point Likert scale) for First Nations mothers. Meanwhile, Routledge et al [81] found that Strong & Deadly Futures (a web-based lesson package aiming to improve mental well-being and substance use prevention) was effective in 53.3% of First Nations students, who were likely to use the knowledge they obtained from the course, and no participants reported that they were unlikely to apply that knowledge. Moreover, 73.3%, 66.7%, and 80.0% of First Nations students thought that this program was helpful in managing substance use, stress, and peer pressure, respectively. However, Dingwall et al [68] found mixed therapeutic outcomes for the AIMhi-Y general mental health support app among First Nations youth. More specifically, the app was found to be effective in reducing psychological distress and depressive symptoms (Cohen *d*=0.85 and 0.75, respectively) and reducing anxiety symptoms (without statistical significance), but was not effective in managing substance use disorders or improving psychological adjustment. Furthermore, Tighe et al [84] found that the iBobbly app was not effective in managing suicidal ideation and reducing psychological distress or depression. However, all indicators presented a trend of improvement. See Table 1 and Multimedia Appendix 3 for detailed information.

#### Quantitative Evidence With Comparison

Five studies examined the effectiveness of DMH services in managing [85,86] and assessing [72,82,87] mental health conditions by comparing the effectiveness of DMH services with that of typical mental health services [72,82,87] or waitlist control groups [85], or comparing the effectiveness of DMH services between First Nations people and non-First Nations people [86].

The Grog Survey app [72], which was developed for assessing substance abuse among First Nations people, was found to be effective in detecting the alcohol consumption level (94.7% overall agreement between the app and clinical interviews). The app was also found to be more sensitive than a clinical interview for the number of drinks, the greatest number of drinks, the median of reported drinking, and the daily consumption of standard drinks per drinking occasion (mean difference=1.6, 0.8, 3, and 0.4, respectively). Higher sensitivity was found among those classified as risky drinkers (2.3% and 4.5% more detection among males and females, respectively), as well as in the reporting (17.0% vs 7.2%) and prediction (*P*=.02 vs *P*=.44) of tremors. See Table 1 and Multimedia Appendix 3 for detailed information.

A previous study compared the telehealth versions of the Mini-Mental State Examination (TMMSE) and Kimberly Indigenous Cognitive Examination-short form (TKICA-screen), which were used for cognitive screening, with their typical versions [87]. The study found that the median TMMSE scores were slightly lower than the Mini-Mental State Examination (MMSE) scores (median difference=4.5), and the agreement measured by correlation was moderate (*r*_s_=0.33; *P*=.20) but with unacceptable limits of agreement (the effectiveness may not significantly reduce in the TMMSE). On the other hand, the TKICA-screen showed a significant effectiveness loss compared with the Kimberly Indigenous Cognitive Examination-short form (KICA-screen). For instance, there was a significant mean difference of 2.17, with a tendency for poorer agreement at the lower end of the performance. See Table 1 and Multimedia Appendix 3 for evidence of proportional bias. Russel et al [82] found that the KICA-screen delivered through digital platforms was as effective as the face-to-face version, with a statistically significant correlation (Pearson *r*=0.851; *P*<.01) and agreement (intraclass correlation coefficient=0.85; *P*<.01) between the versions.

Titov et al [86] found that the MindSpot service, aiming to provide general mental health support, was effective in managing psychological distress, depression, and anxiety, with statistically significant decreases in the Kessler Psychological Distress Scale-10, Patient Health Questionnaire-9, and Generalized Anxiety Disorder Scale-7 scores (*χ*^2^=5933.1, 5938.8, and 5658.8, respectively). As a DMH service designed for all Australians, there were no statistically significant differences in treatment outcomes between First Nations patients and non-First Nations patients or between First Nations patients who chose the general well-being course and those who chose the First Nations well-being course.

Tighe et al [85] evaluated the iBobbly app that was designed for suicide prevention. The results suggested that there were no statistically significant differences between the iBobbly app group and the waitlist control group in suicide prevention (*t*_58.1_=2.40; *P*>.05). However, it was effective in managing depression when compared with a waitlist control group and preintervention baseline scores (*P*<.05). Similarly, the DMH service was found to be effective in managing psychological distress (*t*_57.5_=2.44). See Table 1 and Multimedia Appendix 3 for *P* values and effect sizes. The iBobbly app was found to not be effective in managing impulsivity.

### Determinants of DMH Service Implementation

#### Overview

Overall, this review identified the determinants of facilitators and barriers that influence the implementation of DMH services. See Table 3 for the extracted themes.

Table 3 presents 9 themes and 26 subthemes that describe the facilitators and barriers influencing the implementation of DMH services for First Nations people. There were 4 subthemes under *organizational factors*, 4 subthemes under *cultural appropriateness*, 6 subthemes under *accessibility*, 1 subtheme under *engagement*, 2 subthemes under *coverage*, 6 subthemes under *acceptability*, 1 subtheme under *integration of DMH services with existing context*, 4 subthemes under *digital literacy*, and 1 subtheme under *efficiency of DMH services*.

**Table 3.** Themes and subthemes of the identified studies.

Theme and subtheme		Supporting evidence
Theme 1: Organizational factors within DMH^a^ service providers		
Supportive structure and managers within DMH service provider organizations facilitate DMH service uptake		Supportive structures or working culture within organizations facilitated resource allocation or staff training for DMH service uptake [78-80]. Enthusiastic managers provided direct support, which increased the willingness of staff and the organization itself for DMH service uptake [66,80].
Policy and structural obstacles and a lack of resources in DMH service provider organizations create barriers for DMH service uptake		Procedural and administrative obstacles and obstructive IT policies limited the access of staff to technologies and DMH services and created failures in digital resource allocation [66,67,79,80]. High workloads within the workplace and a lack of digital resources resulted in a lack of DMH training for DMH service providers [66,79,80].
Theme 2: Cultural appropriateness of DMH services		
DMH services could be culturally appropriate owing to cultural safety designs and culturally relevant content in DMH services and empowerment, which can increase the willingness of First Nations people to use DMH services		Overall cultural safety reported by participants [74]. DMH services empowered clients to record their own information and interact with service providers [78,80]. Cultural safety designs were applied in DMH services, which created a safe environment or directly increased the acceptability and interest of clients [69,70,75,76,83,84].
Lack of cultural appropriateness creates barriers for First Nations users to overcome existing negative perceptions and accept DMH services		Negative perception toward the health system created barriers for First Nations people to use DMH services [67]. Problematic use of English within DMH services and lack of First Nations languages may cause difficulties in using DMH services [75,83].
Theme 3: Accessibility of First Nations people to DMH services		
First Nations people have mixed perceptions of their accessibility to DMH services		First Nations clients generally appraised the high accessibility of DMH services, such as reporting that the DMH services they received were accessible [83,86]. However, general accessibility-related concerns, such as a lack of services and resources, and long response times, were also expressed [67].
Portability and flexibility of DMH services, as well as existing digital infrastructure coverage among First Nations communities, could facilitate the implementation of DMH services		DMH services were available on multiple digital platforms [75]. A high coverage of digital infrastructure among communities was presented, which facilitated the uptake of DMH services [67,77]. DMH services can be operated on portable devices, which can create unique advantages in accessibility [78,84].
Many First Nations people report limited access to appropriate digital infrastructure, resulting in low accessibility to DMH services		In some First Nations communities, a lack of digital infrastructure and devices resulted in difficulties in the implementation of DMH services [69,77-80]. Even in special environments, such as prisons, access to digital devices and some specific functions of DMH services, such as access to images, was limited [74]. In remote areas, a lack of digital infrastructure created barriers for First Nations residents to implement DMH services [75,78].
Free DMH apps are highly accessible, while those with download fees limit client access		Apps with high accessibility were preferred, such as free-to-download apps that can operate on different platforms [76]. The cost of e-mental health apps negatively influenced their accessibility [75].
DMH services that do not have First Nations language versions create accessibility barriers for First Nations people whose first language is not English		A lack of DMH services in First Nations languages created accessibility barriers [69,75,78].
Theme 4: Ability of DMH services to promote engagement		
DMH services can promote engagement between clients and service providers by breaking the power imbalance and then creating facilitating effects		DMH services could help establish positive relationships between health care providers and clients, eventually creating positive experiences for clients or increasing First Nations clients’ willingness to use these services [69,74,78].
Theme 5: Ability of DMH services to cover a wide range of settings and First Nations population groups		
DMH services could cover different types of clients, topics, and reference periods		High applicability across different age groups and mental health conditions [69]. DMH services could cover sensitive topics due to their nature of indirect engagement [69,78]. Compared to typical face-to-face mental health services, DMH services could cover longer reference periods on substance use topics [72].
DMH services fail to cover some severe mental health conditions, which is considered a barrier		DMH services may not be able to cover severe mental health conditions, such as suicide [75,78].
Theme 6: Acceptability of First Nations people to DMH services		
Acceptability of First Nations people to DMH services is perceived as high		Satisfaction and optimism regarding DMH services and willingness to use DMH services to seek help were presented by First Nations clients, indicating high acceptability [68,69,71,74,75,77,81,84,86]. First Nations people also expressed their idea of not expecting to receive help from DMH services [73].
Good visual design can facilitate participants’ acceptance of DMH services, while visual design problems are considered barriers		Clients reported that the DMH services they received had an attractive or engaging visual design, which increased their acceptability of DMH services [68,69,71,74,78]. Some visual design problems also raised the attention of clients, such as high text density [70].
The unique nature of DMH services has high acceptability among First Nations people		First Nations clients preferred the web-based, client-led, and clinician-supported nature of DMH services [74,75,83].
Attractive and useful content included in DMH services facilitates First Nations people to accept DMH services		First Nations clients perceived that mental health resources in DMH services were generally interactive and useful [71,80]. Embedded media forms, such as games, videos, and animations, were especially preferred by First Nations clients [68,75]. Tailored content for First Nations students was welcomed [83].
Inadequate, repetitive, and less engaging content included in DMH services is considered a barrier		Inadequate and repetitive content in DMH services reduced the acceptability of DMH services [68,71,84].
The unique functions of DMH services increase their acceptability among First Nations clients		The unique functions of DMH services, such as text messages sent as reminders or data capture functions, were acceptable for First Nations clients and prompted clients to use those services [68,69,74,78].
Some functions are not attractive enough or are difficult to use, resulting in lower acceptability of DMH services		However, some functions of DMH services were ignored, could hardly be used, or could not fit in special contexts, such as in prison [68,71,74,75].
DMH services with data safety have high acceptability, but the concern about data safety is perceived as a barrier		DMH service apps with high privacy and data safety were preferred [76]. Some service providers and clients also considered data unsafety as a potential risk and barrier [67,78].
Theme 7: Ability of DMH services to integrate with existing contexts		
Some DMH services are hard to integrate with existing contexts		Some service providers felt that it was challenging to integrate DMH services into their routine care because DMH services may not easily fit group clients, pre-existing practices, and local community status [79,80].
Theme 8: Digital literacy of clients and service providers		
Clients and service providers who have high digital literacy facilitate the uptake of DMH services		Young clients presented especially high digital literacy and willingness to receive DMH services [67,80]. Based on their own enthusiasm for using DMH services or specific DMH training, some service providers obtained high DMH literacy [66,67].
Clients who do not have high digital literacy may face challenges when operating DMH services		Some clients reported that the DMH app is difficult to operate [70,75,78]. Relatively lower digital literacy resulted in lower awareness of DMH services [68,75,80].
Service providers who do not have high digital literacy may face challenges when operating or providing DMH services		Low digital literacy of service providers led to negative perceptions, including overestimating the complexity of technology or thinking DMH services will distract them from their current job [66,67,78]. Inadequate digital literacy among service providers created challenges in maintaining and using DMH services [67,79,80]. Overall, the digital literacy level among service providers was highly heterogeneous [78,79].
Theme 9: Efficiency of DMH services		
DMH services have high efficiency and are considered time-saving		Service providers believed that DMH services could reduce the time cost of recording client notes and waiting for data transmission, and these had advantages for data recording and management compared to typical paper format services [78,80].

a DMH: digital mental health.

#### Organizational Factors

Organizational factors related to the administration and management of the organization (usually service providers) could either facilitate or create barriers to implementing DMH services.

Four studies asked stakeholders and service providers to generally comment on DMH services [66,78-80]. The supportive structure within the organizations was a significant facilitator, which included the regular supervision and review of DMH service utilization [78]; being supportive of allocating resources, time, and training [79]; and building the work culture that welcomes DMH services within the workforce [80]. Direct support from managers was also considered to facilitate the uptake of DMH services [80] by increasing the opportunity and interest of staff in adopting DMH services [66].

Four studies asked mental health service providers about their general perspectives on DMH services [66,79,80] and on using DMH services in suicide prevention [67]. Service providers reported that organizational factors created barriers for the uptake of DMH services. These included a lack of organizational support [80], administrative obstacles, obstructive IT policies within the workplace [66,79], work restrictions [67], a lack of training resources within organizations [79], and time allocated to cope with the extra workload brought on by the implementation of DMH services [66,79,80].

#### Cultural Appropriateness

Cultural appropriateness was perceived as a critical determinant of the uptake of DMH services within First Nations people in Australia, with high cultural appropriateness facilitating uptake and low cultural appropriateness impeding uptake.

Ten studies reported high culturally appropriate DMH services [69-71,74-76,78,81,83,84]. The users of the AIMhi Stay Strong app [69] and its custodial version [74] reported satisfaction with cultural safety. Similarly, participants in programs, such as the “SMS4dads” mobile app program, “Stayin’ on Track” website program [70], AIMhi-Y app program [76], and iBobbly suicide prevention program [84], valued the culturally appropriate visual and content design [76] as well as the private space these tools provide to reduce shame and stigma [75,84]. Stakeholders also highlighted the empowering nature of DMH services that allowed users to record personal information and engage with culturally meaningful content [71,78].

The “Strong & Deadly Futures” web-based substance use prevention program, though designed for the general population, was also praised for its cultural appropriateness owing to its inclusion of First Nations characters and an equitable learning environment for both First Nations and non-First Nations students [81,83].

Three studies reported a lack of cultural safety within DMH services [67,75,83]. Some First Nations health workers perceived digital suicide prevention tools as culturally inappropriate, reflecting broader concerns about cultural unsafety in the mental health system [67]. Users of the AIMhi Stay Strong app and the iBobbly suicide prevention app expressed uncertainty about whether these tools could address trauma linked to colonization [75], and “Strong & Deadly Futures” participants provided mixed responses for the use of “Aboriginal English” [83].

#### Accessibility

Accessibility was another key determinant of DMH service implementation among First Nations people. Two studies generally evaluated accessibility [83,86], noting that MindSpot could deliver care when local face-to-face mental health services were unavailable [86], and Strong & Deadly Futures was accessible to users with varying abilities [83]. However, concerns remained about poor communication systems, limited resources, slow response times, and service gaps in digital suicide prevention tools [67].

Five studies reported that improved digital infrastructure supported access to DMH services [67,75,77,78,84]. Apps, such as AIMhi and iBobbly, were considered accessible due to their compatibility with multiple devices and their portability [67,75,77,78,84], which enabled use across locations and levels of remoteness. Nevertheless, limited availability of devices and internet connectivity reduced access [78-80], particularly in remote communities and custodial settings [74,75].

Cost was another barrier, with participants preferring free-to-download apps, and the fees for the AIMhi Stay Strong and iBobbly apps were seen as deterrents [75,76].

Finally, language barriers restricted access, as most apps lacked First Nations language options, making it difficult for some users to understand key terms such as “resilience” [69,75,78].

#### Integration

The difficulty in integrating DMH services with existing practices was generally perceived as a barrier in the included studies. More specifically, some service providers in 2 studies commented on applying DMH services in general mental health areas [79,80] and reported difficulties in integrating DMH services into usual practices when facing non-one-to-one settings, such as facing a group of clients [79], or the need to fit existing practices and the local community status [80].

#### Engagement (Between Service Providers and Clients)

Three studies highlighted that DMH services promoting engagement between clients and service providers were well received in general mental health care [69,74,78]. The AIMhi app was reported by health providers to reduce barriers, such as power imbalance, and foster positive relationships between health care providers and clients [69]. Moreover, stakeholders expanded this result to other DMH tools that could provide indirect engagements when solving sensitive issues and supported the building of positive relationships [78]. Improved rapport was also observed in custodial settings through the custodial version of the Stay Strong app [74].

#### Coverage

Coverage refers to the ability of DMH services to cover different types of clients and various mental health conditions. Three studies found that broader coverage facilitated implementation [69,72,78]. The AIMhi app was reported to reach clients across age groups and symptom profiles, including those who were otherwise difficult to engage [69]. Moreover, clients of the AIMhi app [69] and service providers providing general opinions on DMH services [78] noted that DMH services could address sensitive mental health topics. Furthermore, the Grog Survey app, which aimed to investigate the alcohol dependence status, enabled coverage of longer reporting periods than typical clinical interviews [72].

However, 2 studies reported that clients and service providers felt DMH services, including the AIMhi and iBobbly apps, may be unsuitable for addressing severe mental health conditions such as suicide [75,78].

#### Acceptability

Acceptability refers to the extent to which DMH services are acceptable to First Nations Australians. Ten studies mentioned overall positive acceptability [68,69,71,73-75,77,81,84,86]. Except for Lifeline Australia, which was rated low due to participants’ limited expectations of receiving suicide prevention support [73], all other services, including the AIMhi Stay Strong app and its custodial and youth versions [68,69,74], “MAMA-EMPOWER” [71], AIMhi, iBobbly [75,84], “Strong & Deadly Futures” [81], and MindSpot [86], were generally well received, showing high satisfaction, usability, and acceptability [77].

The nature of DMH services was reported as acceptable by 3 studies [74,75,83]. The custodial version of the AIMhi app was appraised for its client-led nature [74], and the AIMhi app and iBobbly suicide prevention app were praised for their clinician-supported nature that could overcome mental health literacy barriers [75]. The web-based nature of “Strong & Deadly Futures” was preferred by students of web lessons [83].

Two studies emphasized relevance and noted that MAMA-EMPOWER was meaningful to users facing relevant issues, although substance use content was viewed as less relevant by nonusers or younger students [71,81].

Four studies discussed digital functions, identifying useful features, such as text messaging in AIMhi [68], service consistency functions in AIMhi [69], goal-setting in the custodial Stay Strong app [74], and data capture features [78]. However, some functions were ineffective or difficult to use, such as the “stories” page in AIMhi-Y [68], and had limited avatar customization [74] and hard-to-use designs [71,75].

Five studies described content as a major contributor to acceptability [68,71,75,80,83]. Participants valued engaging and informative material, including media components (games, videos, and graphics) and culturally tailored content for First Nations users [83]. Conversely, inadequate or repetitive content reduced engagement [68,71,84].

Six studies addressed visual design [68-71,74,78]. Most participants praised the cultural appropriateness, color, and user-friendly layout of AIMhi and MAMA-EMPOWER, while some found SMS4dads and Stayin’ on Track overly text-heavy [70]

Finally, 3 studies discussed data safety and privacy as concerns [67,76,78]. Youth participants preferred DMH tools with secure data handling [76], but general apprehension about data security, particularly in suicide prevention contexts, was reported as a barrier [67,78].

#### Digital Literacy

Digital literacy was identified as a key determinant of the implementation of DMH services. Three studies reported that high digital literacy facilitated the use and acceptability of DMH services [66,67,80]. Young First Nations clients with higher digital literacy demonstrated greater acceptance of DMH tools [67,80], and some suicide prevention gatekeepers preferred digital platforms, such as Facebook, for service delivery [67]. Training also improved providers’ digital literacy, enhancing DMH adoption [66].

However, low digital literacy hindered engagement for both clients [68,70,75,78,80] and service providers [66,67,78-80]. Clients reported difficulty operating apps, such as SMS4dads, Stayin’ on Track, AIMhi, and iBobbly [70,75,78], with some forgetting or being unaware of DMH tools [75]. Among providers, digital competency varied widely, with some describing themselves as “less technologically competent” and struggling with basic functions like downloading apps [67,79,80].

#### Efficiency

Efficiency was another determinant of the implementation of DMH services. Two studies [78,80] reported that service providers perceived DMH services as improving efficiency. More specifically, DMH services could reduce the time cost of recording client notes and waiting for data transmission [78], offering advantages in data recording and management compared with traditional paper-based systems [80].

## Discussion

### Principal Findings

This is the first review to synthesize evidence on the effectiveness of DMH services and the determinants of the facilitators and barriers of DMH services for First Nations people in Australia. Our review found that DMH services were effective in assessing mental health conditions, monitoring mental health status, and delivering mental health education, and had the potential to improve mental health conditions among First Nations Australians. However, the effectiveness of these services for severe mental health conditions remains limited. Additionally, this review identified determinants that could facilitate or inhibit the uptake of DMH services among First Nations people in Australia.

### Effectiveness of DMH Services

The effectiveness of DMH services for First Nations Australians varied across different mental health outcomes and intervention types. Consistent with a previous government report [88] and similar studies [89-91], qualitative studies included in this review suggested that DMH interventions can support mental health improvement, increase self-reflection, and enhance self-confidence and empowerment [68,74,75,80,81,84].

Considering that most included DMH services applied culturally adapted designs, these findings align with broader evidence that culturally adapted DMH tools can provide valuable support for the mental health of First Nations people [3,10,92]. However, concerns remain about the adequacy of digital interventions for severe mental health conditions, particularly suicide prevention, where some participants expressed doubts about their effectiveness beyond mild to moderate distress [75,84,85]. This highlights the ongoing challenge of delivering digital suicide prevention interventions effectively in First Nations communities, as also noted in previous research [55].

Moreover, evidence supports the effectiveness of DMH interventions for substance use. The Grog Survey app accurately identified at-risk drinkers with high sensitivity and reliability [72], and it was as effective as the web-based lesson package Strong & Deadly Futures in preventing substance use [81]. However, the AIMhi-Y app was ineffective in reducing alcohol and drug use symptoms [68], indicating that while DMH tools could be valuable for monitoring and performing early intervention, they might not have sufficient therapeutic impact for substance use disorders without additional face-to-face support. This aligns with previous research on First Nations people in the United States, who had insignificant therapeutic outcomes [93]. The use of DMH interventions for cognitive assessment has also yielded mixed results [82,87]. These findings highlight the need for careful adaptation of cognitive assessments for telehealth delivery, as the telehealth version of the test reduced the visual cues of the test [87] and resulted in insignificant results, but the same test that was delivered through video conferencing obtained acceptable effectiveness compared with typical in-person services [82]. Finally, our engagement and user perception findings indicated that DMH education and behavioral change interventions can be beneficial but require further refinement. For instance, the Strong & Deadly Futures program was generally acceptable to First Nations students owing to the perceived effectiveness in preventing substance use [81]. However, a substantial proportion of students remained unsure about applying the information in their own lives. This suggests that while digital education programs can improve mental health literacy, their impact on long-term behavioral change may be limited without additional reinforcement, as noted in previous research [94].

This review found that the effectiveness of DMH services for First Nations people in Australia is in line with that for First Nations people from other countries. For example, the effectiveness of DMH services in Australia was found in international evidence, such as a general mental health program that successfully caused positive behavioral change for First Nations people in Canada [95] and a mental health screening program in New Zealand [96]. This indicates that the experience and knowledge generated from First Nations people can be generalized. Heterogeneity was present in some areas. For example, a program in the United States achieved statistical significance in treating substance use issues for First Nations people [93], while a program with a similar target only obtained insignificant results in Australia [68]. Considering that the situation and historical factors faced by First Nations people in Australia are unique, care should be taken when generalizing the results from this review.

### Determinants of the Uptake of DMH Services Among First Nations People in Australia

This review highlights that the implementation of DMH services for First Nations people is shaped by interconnected structural, cultural, and individual factors. Organizational readiness, including leadership support, workforce capacity, and digital infrastructure, remains foundational. Leadership drives the strategic vision, fosters innovation, and ensures resource allocation, which are essential for successful implementation [97,98]. Strong leadership also facilitates stakeholder engagement and addresses resistance to change, which are key factors in digital health uptake [99]. Organizational support further enhances workforce readiness and policy development, sustaining long-term adoption [100]. Without these elements, DMH initiatives risk poor adoption and limited impact [66,67,79,80]. Additionally, cultural appropriateness emerged as a defining determinant of uptake. Culturally safe, co-designed tools enhanced engagement and empowerment, whereas culturally unsafe or linguistically limited designs hindered trust and acceptability. Culturally relevant app designs incorporating First Nations art and metaphors were found to enhance engagement [69,70,75,76,83,84]. This echoes prior research emphasizing the importance of embedding cultural elements to ensure relevance and acceptance within First Nations communities [101].

Accessibility, digital literacy, and efficiency collectively reflect the broader digital divide that continues to shape health equity in Australia. First, inconsistent digital literacy among both clients and practitioners has been previously identified as a key obstacle to effective use of DMH services [66-68,70,75,78-80]. Delivering technology training and ongoing support could improve digital literacy and confidence in using DMH services [102,103], ultimately enhancing their uptake and efficacy among First Nations communities. Second, while improved infrastructure and targeted training enhanced access and usability in some First Nations communities, persistent disparities in internet connectivity, device availability, and digital skills constrained the scalability of DMH interventions, particularly in remote and custodial settings [104]. Despite evidence showing common ownership of digital infrastructure in some First Nations communities [67,77], limited infrastructure and technology access remain significant challenges, particularly in remote communities where access to tablets, Wi-Fi, and reliable technology is often restricted [67,69,75,77-80]. It has been reported that First Nations Australians, especially those living in remote areas, are some of the most digitally excluded populations [105]. This finding highlights the urgent need for targeted investments in digital infrastructure to bridge the technology gap and ensure equitable access to DMH services. To address these challenges, it could be helpful to expand community Wi-Fi programs, provide subsidized digital devices, and implement mobile-based DMH solutions that require minimal bandwidth [88]. The Mob Link initiative by the Institute for Urban Indigenous Health is a strong example [106], as it provides culturally responsive digital health support, including assistance with telehealth and digital access, helping to reduce barriers and improve engagement with health care services. Without sustained investment in digital inclusion and community-based training, DMH services risk reproducing existing inequities rather than closing service gaps [107].

At the service level, DMH tools enhance engagement and communication between clients and providers, supporting self-reflection and rapport. However, limited integration with existing care models, concerns about data privacy, and uncertainty about applicability to severe mental health conditions reduce trust and engagement with DMH services and signal the need for clearer clinical pathways and blended-care frameworks. For instance, privacy concerns and the fear of data misuse have been constantly identified as significant deterrents to the uptake of DMH services [67,78]. It is imperative to address the concerns of First Nations people regarding data sovereignty and the potential misuse of personal health information [108], so that DMH services can be designed with robust privacy protection and transparent data governance frameworks. Furthermore, streamlined data management and integration may be helpful for integrating DMH services and reducing administrative burden, enabling system-level adoption of DMH services for First Nations Australians.

### Heterogeneity of DMH Service Types

This review included various DMH service types, which caused differentiation in determinants. For example, the telehealth version of an included cognitive screening tool showed lower effectiveness due to the absence of visual cues in the test [87], while the same test with visual cues obtained acceptable effectiveness [82]. A possible explanation for this difference is that the latter test was delivered through video conferencing and involved a complete version of the assessment containing images and visual cues. Similarly, the visual design and multimedia content of DMH apps [68,69,71,74,75,78,80,83] and the various digital functions in DMH apps [68,69,74,78] were praised for increasing the acceptability of DMH services among First Nations people. However, no similar facilitators were reported by the users of telehealth DMH services, hinting at the potential benefits of designing app- or web-based DMH services compared with telehealth DMH services. Despite such diversities, this review aimed to synthesize all evidence representing technology-assisted mental health services. Therefore, it has provided an integrated understanding of how digital approaches have been applied to support mental health among First Nations people.

### Limitations

We acknowledge that this review has several limitations. First, the included studies were highly heterogeneous in terms of intervention types, study designs, and outcome measures. This limited our ability to draw precise or generalizable conclusions about overall effectiveness. Second, as our search was restricted to academic databases, relevant gray literature may have been missed, potentially contributing to publication bias. Third, many included studies had a moderate to high risk of bias, with small sample sizes and self-reported outcomes, which may reduce the certainty of the evidence.

### Conclusion

This review highlights the effectiveness of DMH services in assessing, monitoring, and managing mental health conditions for First Nations Australians. However, information on their impact on severe conditions, including suicide, remains limited. Digital health apps showed promising results in improving general mental health support. Digital health apps and online courses were also perceived to be effective in preventing and assessing substance use, but did not have a significant treatment effect on managing it. For screening mental health conditions, the digital health versions of instruments were comparable to face-to-face versions, while the telehealth versions showed less effectiveness than both the digital health and face-to-face versions, indicating that effectiveness may vary across different types of DMH services.

Long-term funding in DMH services through culturally responsive community services (eg, Institute for Urban Indigenous Health) from the government or organizations should be beneficial, especially for First Nations people facing barriers to accessing in-person mental health services (eg, those who reside in remote areas) and for DMH services that are supported by evidence.

This study also identified key facilitators (eg, strong leadership, culturally relevant designs, clinician-supported tools, and community) and major barriers (eg, digital exclusion, low digital literacy, and privacy concerns). These findings highlight the need for targeted infrastructure investment that can reduce the digital divide for First Nations people, especially in regional and remote communities; digital training for both mental health care providers and First Nations clients; and transparent data governance. However, the evidence should be interpreted with caution, given the heterogeneity of study designs and outcomes, potential publication bias, and moderate to high risk of bias across the included studies. Future research should focus on co-designing DMH services with First Nations communities, rigorously evaluating their effectiveness, and integrating them into existing health care models to ensure accessibility, cultural relevance, and long-term impact.

## Supplementary material

10.2196/80386 Multimedia Appendix 1 Detailed inclusion and exclusion criteria, and PICO (population, intervention, comparison, outcome) protocol.

10.2196/80386 Multimedia Appendix 2 Details of the search strategy.

10.2196/80386 Multimedia Appendix 3 Details of the extracted data.

10.2196/80386 Multimedia Appendix 4 Quality assessment results (Mixed Methods Appraisal Tool).

10.2196/80386 Multimedia Appendix 5 Quality assessment results (Aboriginal and Torres Strait Islander Quality Appraisal Tool).

10.2196/80386 Checklist 1 PRISMA checklist.

## Abbreviations

AIMhi: Aboriginal and Islander Mental Health Initiative
DMH: digital mental health
KICA-screen: Kimberly Indigenous Cognitive Examination-short form
MMAT: Mixed Methods Appraisal Tool
MMSE: Mini-Mental State Examination
PICO: population, intervention, comparison, outcome
PRISMA: Preferred Reporting Items for Systematic Reviews and Meta-Analyses
TKICA-screen: telehealth version of the Kimberly Indigenous Cognitive Examination-short form
TMMSE: telehealth version of the Mini-Mental State Examination
