# Supplementary material for: The Effectiveness, Facilitators, and Barriers of Digital Mental Health Services for First Nations People in Australia: Systematic Scoping Review
Source: Interact J Med Res. 2026 Jan 27;15:e80386. doi: 10.2196/80386 (PMC12841858; doi:10.2196/80386)
Supplement: Multimedia Appendix 1 [file ijmr-v15-e80386-s001.docx]

| Patient/Population | Intervention | Comparison | Outcomes |
| --- | --- | --- | --- |
| - First Nations Peoples in Australia who have mental health conditions or are at risk of having mental health conditions. | - Digital Mental Health Services, including:  - Screening, diagnosis and treatment towards mental illness.  - Prevention and support for mental health issues, including suicide or self-harm behaviours and substance use. | - Typical non-digital mental health services  - No comparison | - Accurate diagnosis and screening of mental health conditions  - Relieve or improve symptoms of mental health conditions  - Prevention of mental health conditions. |

| Inclusion Criteria | Exclusion Criteria | Extra Criteria |
| --- | --- | --- |
| - Be written in English - Studies on First Nations Peoples in Australia - Studies on DMH services - Studies on digital health services have a purpose other than mental health but contain DMH services as their components. - Studies on DMH services provided for the general population but specified the data of First Nations Peoples. - Studies on users’ or service providers’ experience of using DMH services. - Studies on determinants affecting the use of DMH services | - Studies on First Nations Peoples outside of Australia. - Studies on Non-First Nations People. - Studies that have not researched digital mental health services. - Studies irrelevant to the determinants affecting the use of DMH services - Review articles, conference papers, research protocols, and other non-primary research studies. - Full text unavailable online. | - No specific limit to the publication dates of studies (DMH is a relatively new area) - No specific limit on the age of participants - No limit on the nation groups of First Nations Peoples in Australia |
