# Supplementary material for: The Effectiveness, Facilitators, and Barriers of Digital Mental Health Services for First Nations People in Australia: Systematic Scoping Review
Source: Interact J Med Res. 2026 Jan 27;15:e80386. doi: 10.2196/80386 (PMC12841858; doi:10.2196/80386)
Supplement: Multimedia Appendix 2 [file ijmr-v15-e80386-s002.docx]

Search terms:

First Nations Peoples (theme):

Indigenous OR Aboriginal OR “First Nations” OR “Torres Strait Islander” OR ATSI

Mental health (theme):

“Mental illness” OR “Psychological disorder” OR “Mental disorder” OR “Mental health” OR “Psychotic Illness” OR “Psychiatric disorder” OR “Mental Condition”

Digital Mental Health (theme):

“Digital health” OR “Digital mental health” OR telemedicine OR telehealth OR ehealth OR e-health OR “electronic health” OR “health apps” OR mhealth OR m-health OR “mobile health” OR “online health” OR “eMental Health” OR “Mental Health Apps” OR Web-based OR mtherapy OR “Online therapy” OR “Online intervention” OR “Online Self-help”

Mental Health Conditions (theme):

Depression OR “Major depressive disorder” OR Dysthymia OR Anxiety OR Schizophrenia OR “Bipolar disorder” OR “Eating Disorder” OR “Anorexia nervosa” OR “Bulimia nervosa” OR “Attention-deficit hyperactivity disorder” OR ADHD OR “Autism Spectrum Disorder” OR “Conduct disorder” OR “Idiopathic developmental intellectual disability” OR Suicide OR Self-harm OR Suicidal

Australia (theme):

Australia* OR Victoria OR Queensland OR Tasmania OR “New South Wales” OR “Western Australia” OR “Northern Territory” OR “Australian Capital Territory” OR “South Australia”

Search Strategy for each database:

PsycINFO: (Indigenous OR Aboriginal OR "First Nations" OR "Torres Strait Islander" OR ATSI) AND ("Mental illness" OR "Psychological disorder" OR "Mental disorder" OR "Mental health" OR "Psychotic Illness" OR "Psychiatric disorder" OR "Mental Condition") AND ("Digital health" OR "Digital mental health" OR telemedicine OR telehealth OR ehealth OR e-health OR "electronic health" OR "health apps" OR mhealth OR m-health OR "mobile health" OR "online health" OR "eMental Health" OR "Mental Health Apps" OR Web-based OR mtherapy OR "Online therapy" OR "Online intervention" OR "Online Self-help") AND (Depression OR "Major depressive disorder" OR Dysthymia OR Anxiety OR Schizophrenia OR "Bipolar disorder" OR "Eating Disorder" OR "Anorexia nervosa" OR "Bulimia nervosa" OR "Attention-deficit hyperactivity disorder" OR ADHD OR "Autism Spectrum Disorder" OR "Conduct disorder" OR "Idiopathic developmental intellectual disability" OR Suicide OR Self-harm OR Suicidal) AND (Australia* OR Victoria OR Queensland OR Tasmania OR "New South Wales" OR "Western Australia" OR "Northern Territory" OR "Australian Capital Territory" OR "South Australia")

PubMed: ((((Indigenous OR Aboriginal OR "First Nations" OR "Torres Strait Islander" OR ATSI) AND ("Mental illness" OR "Psychological disorder" OR "Mental disorder" OR "Mental health" OR "Psychotic Illness" OR "Psychiatric disorder" OR "Mental Condition")) AND ("Digital health" OR "Digital mental health" OR telemedicine OR telehealth OR ehealth OR e-health OR "electronic health" OR "health apps" OR mhealth OR m-health OR "mobile health" OR "online health" OR "eMental Health" OR "Mental Health Apps" OR Web-based OR mtherapy OR "Online therapy" OR "Online intervention" OR "Online Self-help")) AND (Depression OR "Major depressive disorder" OR Dysthymia OR Anxiety OR Schizophrenia OR "Bipolar disorder" OR "Eating Disorder" OR "Anorexia nervosa" OR "Bulimia nervosa" OR "Attention-deficit hyperactivity disorder" OR ADHD OR "Autism Spectrum Disorder" OR "Conduct disorder" OR "Idiopathic developmental intellectual disability" OR Suicide OR Self-harm OR Suicidal)) AND (Australia* OR Victoria OR Queensland OR Tasmania OR "New South Wales" OR "Western Australia" OR "Northern Territory" OR "Australian Capital Territory" OR "South Australia")

Medline: (Indigenous OR Aboriginal OR "First Nations" OR "Torres Strait Islander" OR ATSI) AND ("Mental illness" OR "Psychological disorder" OR "Mental disorder" OR "Mental health" OR "Psychotic Illness" OR "Psychiatric disorder" OR "Mental Condition") AND ("Digital health" OR "Digital mental health" OR telemedicine OR telehealth OR ehealth OR e-health OR "electronic health" OR "health apps" OR mhealth OR m-health OR "mobile health" OR "online health" OR "eMental Health" OR "Mental Health Apps" OR Web-based OR mtherapy OR "Online therapy" OR "Online intervention" OR "Online Self-help") AND (Depression OR "Major depressive disorder" OR Dysthymia OR Anxiety OR Schizophrenia OR "Bipolar disorder" OR "Eating Disorder" OR "Anorexia nervosa" OR "Bulimia nervosa" OR "Attention-deficit hyperactivity disorder" OR ADHD OR "Autism Spectrum Disorder" OR "Conduct disorder" OR "Idiopathic developmental intellectual disability" OR Suicide OR Self-harm OR Suicidal) AND (Australia* OR Victoria OR Queensland OR Tasmania OR "New South Wales" OR "Western Australia" OR "Northern Territory" OR "Australian Capital Territory" OR "South Australia")

Embase: ('indigenous'/exp OR indigenous OR 'aboriginal'/exp OR aboriginal OR 'first nations'/exp OR 'first nations' OR 'torres strait islander'/exp OR 'torres strait islander' OR atsi) AND ('mental illness'/exp OR 'mental illness' OR 'psychological disorder'/exp OR 'psychological disorder' OR 'mental disorder'/exp OR 'mental disorder' OR 'mental health'/exp OR 'mental health' OR 'psychotic illness' OR 'psychiatric disorder'/exp OR 'psychiatric disorder' OR 'mental condition'/exp OR 'mental condition') AND ('digital health'/exp OR 'digital health' OR 'digital mental health'/exp OR 'digital mental health' OR 'telemedicine'/exp OR telemedicine OR 'telehealth'/exp OR telehealth OR 'ehealth'/exp OR ehealth OR 'e health'/exp OR 'e health' OR 'electronic health'/exp OR 'electronic health' OR 'health apps' OR 'mhealth'/exp OR mhealth OR 'm health' OR 'mobile health'/exp OR 'mobile health' OR 'online health' OR 'emental health' OR 'mental health apps' OR 'web based' OR mtherapy OR 'online therapy' OR 'online intervention'/exp OR 'online intervention' OR 'online self-help') AND ('depression'/exp OR depression OR 'major depressive disorder'/exp OR 'major depressive disorder' OR 'dysthymia'/exp OR dysthymia OR 'anxiety'/exp OR anxiety OR 'schizophrenia'/exp OR schizophrenia OR 'bipolar disorder'/exp OR 'bipolar disorder' OR 'eating disorder'/exp OR 'eating disorder' OR 'anorexia nervosa'/exp OR 'anorexia nervosa' OR 'bulimia nervosa'/exp OR 'bulimia nervosa' OR 'attention-deficit hyperactivity disorder'/exp OR 'attention-deficit hyperactivity disorder' OR 'adhd'/exp OR adhd OR 'autism spectrum disorder'/exp OR 'autism spectrum disorder' OR 'conduct disorder'/exp OR 'conduct disorder' OR 'idiopathic developmental intellectual disability' OR 'suicide'/exp OR suicide OR 'self harm'/exp OR 'self harm' OR suicidal) AND (australia* OR 'victoria'/exp OR victoria OR 'queensland'/exp OR queensland OR 'tasmania'/exp OR tasmania OR 'new south wales'/exp OR 'new south wales' OR 'western australia'/exp OR 'western australia' OR 'northern territory'/exp OR 'northern territory' OR 'australian capital territory'/exp OR 'australian capital territory' OR 'south australia'/exp OR 'south australia')

Web of Science: Indigenous OR Aboriginal OR "First Nations" OR "Torres Strait Islander" OR aisi (All Fields) and "Mental illness" OR "Psychological disorder" OR "Mental disorder" OR "Mental health" OR "Psychotic Illness" OR "Psychiatric disorder" OR "Mental Condition" (All Fields) and "Digital health" OR "Digital mental health" OR telemedicine OR telehealth OR ehealth OR e-health OR "electronic health" OR "health apps" OR mhealth OR m-health OR "mobile health" OR "online health" OR "eMental Health" OR "Mental Health Apps" OR Web-based OR mytherapy OR "Online therapy" OR "Online intervention" OR "Online Self-help" (All Fields) and Depression OR "Major depressive disorder" OR Dysthymia OR Anxiety OR Schizophrenia OR "Bipolar disorder" OR "Eating Disorder" OR "Anorexia nervosa" OR "Bulimia nervosa" OR "Attention-deficit hyperactivity disorder" OR ADHD OR "Autism Spectrum Disorder" OR "Conduct disorder" OR "Idiopathic developmental intellectual disability" OR Suicide OR Self-harm OR Suicidal (All Fields) and Australia* OR Victoria OR Queensland OR Tasmania OR "New South Wales" OR "Australia" OR "Northern Territory" OR "Australian Capital Territory" OR "South Australia" (All Fields)

Google Scholar: ("Indigenous" OR Aboriginal OR "First Nations" OR "Torres Strait Islander" OR ATSI) AND ("Mental illness" OR "Psychological disorder" OR "Mental disorder" OR "Mental health" OR "Psychotic Illness" OR "Psychiatric disorder" OR "Mental Condition") AND ("Digital health" OR "Digital mental health" OR telemedicine OR telehealth OR ehealth OR e-health OR "electronic health" OR "health apps" OR mhealth OR m-health OR "mobile health" OR "online health" OR "Mental Health" OR "Mental Health Apps" OR Web-based OR therapy OR "Online therapy" OR "Online intervention" OR "Online Self-help") AND (Depression OR "Major depressive disorder" OR Dysthymia OR Anxiety OR Schizophrenia OR "Bipolar disorder" OR "Eating Disorder" OR "Anorexia nervosa" OR "Bulimia nervosa" OR ADHD OR "Autism Spectrum Disorder" OR Suicide OR "Self-harm" OR Suicidal) AND (Australia* OR Victoria OR Queensland OR Tasmania OR "New South Wales" OR "Western Australia" OR "Northern Territory" OR "Australian Capital Territory" OR "South Australia")
